# Cage restriction‐induced physical inactivity promotes subsequent hepatic apoptosis during tail suspension in young male rats

**DOI:** 10.14814/phy2.15695

**Published:** 2023-05-24

**Authors:** Shohei Dobashi, Hisashi Naito, Toshinori Yoshihara

**Affiliations:** ^1^ Graduate School of Health and Sports Science Juntendo University Chiba Japan; ^2^ Institute of Health and Sports Science & Medicine Juntendo University Chiba Japan; ^3^ Japan Society for the Promotion of Science Tokyo Japan

**Keywords:** caspase family, fragmented nucleosomes, heat shock protein, narrow living space, sedentary lifestyle, tail suspension

## Abstract

This study investigated the impact of long‐term physical inactivity on hepatic cytoprotective‐ and inflammatory‐related protein expressions in young rats and the subsequent apoptotic response during microgravity stress simulated by tail suspension. Four‐week‐old male Wistar rats were randomly assigned to the control (CT) and physical inactivity (IN) groups. The floor space of the cages provided to the IN group was reduced to half of that provided to the CT group. After 8 weeks, rats in both groups (*n* = 6–7) underwent tail suspension. Their livers were harvested immediately before (0 day) or 1, 3, and 7 days after tail suspension. Levels of hepatic heat shock protein 72 (HSP72), an anti‐apoptotic protein, reduced over 7 days of tail suspension in the IN group than in the CT group (*p* < 0.01). Fragmented nucleosomes in the cytoplasmic fraction of the liver, an apoptotic index, were drastically increased by physical inactivity and tail suspension, and this change was significantly greater after 7 days of tail suspension in the IN group than in the CT group (*p* < 0.01). The apoptotic response was accompanied by the upregulation of pro‐apoptotic proteins (cleaved caspase‐3 and ‐7). Moreover, the levels of other pro‐apoptotic proteins (tumor necrosis factor‐1α and histone deacetylase 5) were also significantly higher in the IN than in the CT group (*p* < 0.05). Our results indicated that 8 weeks of physical inactivity decreased hepatic HSP72 levels and promoted hepatic apoptosis during the subsequent 7 days of tail suspension.

## Graphical abstract

We demonstrated that tail suspension following eight weeks of physical inactivity drastically increased the number of fragmented nucleosomes in the cytoplasmic fraction of the liver. We believe that our study makes a significant contribution to the literature because our findings suggest that decreased HSP72 levels by physical inactivity may promote the subsequent hepatic apoptosis during tail suspension by increasing pro‐apoptotic proteins (cleaved caspase‐3 and ‐7, TNF‐α, and HDACs 4 and 5).

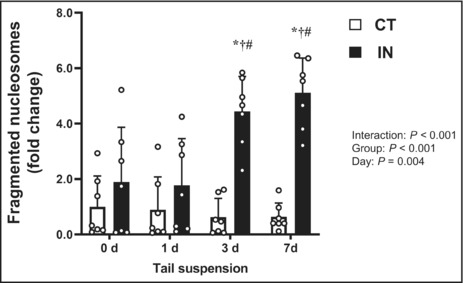

## 1 INTRODUCTION

The liver is an essential organ of the body that performs various physiological functions, including the removal of waste products and foreign substances from the bloodstream, regulation of blood glucose concentrations, and creation of essential nutrients. As hepatic dysfunction induced by inflammation and apoptosis ultimately causes critical liver diseases, such as liver fibrosis, cirrhosis, and the development of hepatocellular carcinoma (Wang & Lin, 2013), attenuation of hepatic damage is a critical step to ensure life‐long hepatic health. Physical activity is well known to induce preventive and therapeutic effects by attenuating inflammation, as well as cell death (apoptosis) in the liver (Zelber‐Sagi et al., 2021). Although the molecular mechanisms underlying this effect are not fully understood, upregulation of the expression of heat shock proteins (HSPs) may be involved. Among them, HSP72 is a stress‐inducible protein, which further induces various cytoprotective properties by assisting with the folding, transport, and degradation of proteins (Balchin et al., 2016). For example, overexpression of hepatic HSP72 protects against apoptosis, oxidative damage, and Jun amino‐terminal kinase signaling induced by a 0.1% 3,5‐diethoxycarbonyl‐1,4‐dihydrocollidine‐supplemented diet (Levada et al., 2018). The major potential inducers of apoptosis are the caspase family and tumor necrosis factor‐α (TNF‐α) (Schwabe & Brenner, 2006; Wang & Lin, 2013), and these molecules are inhibited by the increased expression of HSP72 in hepatocytes (Tang et al., 2007; Tsai et al., 2015). Additionally, growing evidence suggests that chronic exercise upregulates HSP72 levels in the liver (Henstridge et al., 2016). Since early life conditions (environment and events) may have long‐term effects in shaping health and disease vulnerability in later life (Hagemann et al., 2021), the upregulation of hepatic HSP72 expression by regular physical activity during adolescence is important for maintaining life‐long health.

Contrary to the evidence regarding the impact of exercise on health benefits, experimental evidence of ‘physical inactivity’ in young individuals is still lacking. In this context, we recently performed a physical inactivity intervention in young rats using narrow cages with approximately half the usual floor space for 8 weeks (Yoshihara et al., 2021). Our data revealed that physical inactivity for 8 weeks alone did not change the soleus muscle phenotype; however, the soleus muscle weight drastically decreased during 7 days of hindlimb unloading by tail suspension compared with that in age‐matched sedentary control rats. These results suggest that long‐term physical inactivity during adolescence may increase vulnerability to subsequent harmful stress. Tail suspension also causes hepatic damage, including inflammation and apoptosis (Chen et al., 2017; Chen et al., 2020; Du et al., 2015; Rivera et al., 2003); however, no experimental study has assessed the effects of physical inactivity on the liver during adolescence.

Therefore, this study aimed to examine the impact of physical inactivity during adolescence on hepatic HSP72 expression and subsequent stress responses. To this end, we performed 8 weeks of physical inactivity intervention using a narrow cage in young male rats and assessed the hepatic apoptotic responses during the subsequent tail suspension. We hypothesized that long‐term physical inactivity during adolescence reduces hepatic HSP72 levels, which in turn exacerbates hepatic damage during the subsequent tail suspension.

## 2 MATERIALS AND METHODS

### 2.1 Ethics approval

All experimental protocols in this study were approved by the Juntendo University Animal Care Committee (approval number H26‐09) and followed the care and use of laboratory animals set by the Physiological Society of Japan.

### 2.2 Experimental animals and design

The current study presents expanded data from our recent investigation that examined the effect of long‐term physical inactivity on tail suspension‐induced atrophy in rat soleus muscle (Yoshihara et al., 2021). Three‐week‐old male Wistar rats were housed in a climate‐controlled room (temperature: 23 ± 1°C, relative humidity: 55% ± 5%) under 12:12 h light/dark cycle (lights on between 18:00 and 06:00). After a 1‐week acclimation period, the rats were assigned to either a control (CT) group that was housed in a normal cage or a physical inactivity (IN) group that was housed in a cage with restricted usual floor space. All rats had free access to a standard diet (Rodent Lab Diet EQ, 5 L37, LabDiet) and water during the entire duration of the study. Body weights and the food intake of the rats were measured weekly.

### 2.3 Physical inactivity intervention

To reduce physical activity without unnecessary restraint stress, we used a recently established cage‐restriction‐induced physical inactivity model (Yoshihara et al., 2021). Briefly, the rats in the CT group were housed in commonly used normal cages, and the floor space was gradually increased along with the growth of rats (198 cm^2^ for 3‐week‐old rats, 544 cm^2^ for 4‐ to 9‐week‐old rats, 880 cm^2^ for 10‐ to 12‐week‐old rats; 2 rats per cage). The rats in the IN group were housed in a small cage (2 rats per cage) with restricted floor space for 8 weeks (4–12 weeks old) to limit their physical activity. During the 8‐week physical inactivity intervention, the daily spontaneous motor activity of the rats was recorded using a commercial passive infrared sensor detection system (SUPERMEX; Muromachi Kikai Co., Ltd.). Although the floor space in the IN group also increased with the growth of the rats, we adjusted the floor space to limit the physical activity levels in IN rats to almost one‐tenth of that in the CT rats over the 8‐week intervention (Yoshihara et al., 2021).

### 2.4 Tail suspension and liver sampling

All rats were subjected to tail suspension following an 8‐week intervention. The protocol for tail suspension has been previously described (Yoshihara et al., 2019). Briefly, a tail‐cast suspension was applied to each rat, leaving the distal one‐third of the tail free to allow appropriate thermoregulation. The tail cast was attached to a hook on the ceiling of the cage, and the height of the hook was adjusted such that the cast was inclined at an angle of approximately 35° in a head‐down orientation. The rats were free to move around the cage on their front feet. The rats were checked daily for tail lesions or discoloration. Before tail suspension (0 day) and after 1, 3, or 7 days of tail suspension, the rats were anesthetized with isoflurane (3%–5%) and pentobarbital sodium(60 mg·kg^−1^), and liver samples were carefully harvested, frozen in liquid nitrogen, and stored at −80°C until further analysis.

### 2.5 Immunodetection

For western blot analysis, powdered liver (approximately 30 mg) was homogenized in 10 volumes of ice‐cold homogenization buffer (20 mM HEPES [pH 7.4], 150 mM NaCl, 1% [W/v] lithium dodecyl sulfate, and 1% [W/v] Nonidet P‐40) containing cOmplete EDTA‐free and PhosSTOP protease inhibitor cocktails (Roche) using a bead cell disrupter (Microsmash MS‐100, Tomy Seiko Co., Ltd.). The tissue homogenate was centrifuged at 17,000 × *g* for 5 min at 4°C after rotation at 4°C for 1 h. The supernatant was collected and stored at −80°C. Protein concentrations were measured using a bicinchoninic acid (BCA) protein assay kit (Thermo Fisher Scientific). After adjusting the protein concentrations, the appropriate volume of sample was mixed with an equal volume of 2× sample buffer (4% sodium dodecyl sulfate [SDS], 20% glycerol, 10% mercaptoethanol, 0.004% bromophenol blue, and 125 mM Tris–HCl [pH 6.8]), allowing the dilution of total protein concentration to 2 mg/mL. The mixture was boiled at 95°C for 5 min and subsequently stored at −20°C until electrophoresis.

Equal amounts of protein (10 μg) were loaded on 7.5%, 10%, or 12% SDS polyacrylamide gels or 4%–15% TGX polyacrylamide precast gels (Bio‐Rad) and electrophoresed at 150 V for 45–60 min at room temperature (20–25°C). The separated proteins were transferred to polyvinylidene difluoride (PVDF) membranes (Bio‐Rad) at 100 V for 60 min at 4°C. The protein‐transferred membranes were incubated in blocking buffer (3% bovine serum albumin in Tween‐Tris buffer saline [T‐TBS:137 mM NaCl, 20 mM Tris‐HCl, 0.1% Tween 20, pH 7.5]) or PVDF Blocking Reagent from Can Get Signal (Toyobo) for 60 min at room temperature (20–25). After three washes in T‐TBS, the membranes were incubated overnight at 4°C with primary antibodies: HSP72 (1:2000; ADI‐SPA‐810; Enzo Life Science), TNF‐α (1:1000; ab6671; Abcam), cleaved caspase‐3 (1:2000; #96625; Cell Signaling Technology), cleaved caspase‐7 (1:2000; #5625; Cell Signaling Technology), histone deacetylase (HDAC) 4 (1:2000; #7628; Cell Signaling Technology), p62 (1:2000; PM045; Medical Biological Laboratories), and microtubule‐associated protein 1 light chain 3 (LC3, 1:5000; #4108; Cell Signaling Technology). After several washes in T‐TBS, the membranes were incubated with anti‐rabbit (1:10000; #7474; Cell Signaling Technology) or mouse (1:10000; #7076; Cell Signaling Technology) horseradish peroxidase‐conjugated secondary antibodies for 1 h at room temperature (20–25°C). Following several washes in T‐TBS, protein bands were visualized using enhanced chemiluminescence prime reagent (Amersham), and the signal was recorded using a ChemiDoc Touch imaging system (Bio‐Rad). The signal intensity was analyzed using Image Lab v.5.2.1 software (Bio‐Rad). Protein expression levels were normalized to the total protein loaded and stained using Revert 700 Total Protein Stain (LI‐COR Biosciences).

### 2.6 Detection of apoptosis

We assessed hepatic fragmented nucleosomes as an apoptotic index using a commercial enzyme‐linked immunosorbent assay (ELISA) kit (Cell Death Detection kit, Roche) following a protocol modified from a previous study (Mach et al., 2015). To assess the fragmented nucleosomes in the cytoplasmic fraction of the liver, a portion of the liver samples (approximately 30 mg) was homogenized in 250 μL of ice‐cold homogenized buffer (20 mM HEPES, pH 7.4, 4 mM EGTA, 0.1 mM EDTA, 10 mM MgCl_2_) containing cOmplete EDTA‐free protease inhibitor cocktails using Microsmash MS‐100. The tissue homogenate was centrifuged at 900 × g for 5 min at 4°C, and the supernatant was subsequently collected. The protein concentration of each supernatant was determined using the BCA protein assay (Thermo Fisher Scientific). In a preliminary test, we assumed that a 0.12 mg/mL hepatic supernatant was optimal for detecting fragmented nucleosomes using this kit. Thus, the hepatic supernatant was diluted to adjust equal protein concentrations (0.12 mg/mL) using the incubation buffer in the ELISA kit. The subsequent experimental procedures were performed according to the manufacturer's instructions.

### 2.7 Statistical analyses

All statistical analyses were performed using Prism v. 9.0 (GraphPad Inc.). We performed a Kolmogorov–Smirnov test to assess normality for all variables. The results indicated that the values of HSP72, HDACs 4 and 5, p62, and LC‐3II/I ratio did not show normal distribution. Previous studies have demonstrated that statistical results are acceptable assuming the robustness of the analysis of variance (ANOVA) under the situation (Alves et al., 2020; Blanca et al., 2017). Accordingly, we performed Levene's test for the non‐normal data and confirmed the homogeneity of variance. Therefore, two‐way (group × day) ANOVA, followed by Tukey's multiple comparison test, was performed on all variables, and the results were accepted as the statistical basis for interpreting the findings of this study. Moreover, the correlations between hepatic HSP72 and pro‐apoptotic protein (i.e., cleaved caspases‐3 and ‐7, TNF‐α, and HDACs 4 and 5) expression levels, and hepatic fragmented nucleosomes were analyzed using Spearman's correlation analysis. Statistical significance was set at *p* < 0.05. All data are presented as the mean ± standard deviation (SD).

## 3 RESULTS

### 3.1 Liver weight

The liver weights before (0 day) and after 3 and 7 days of tail suspension were previously described (Yoshihara et al., 2021). Although significant effects for each group and day were observed, the multiple comparisons did not show any significant difference in the liver weights between the CT and IN groups for each day examined.

### 3.2 HSP72 expression levels

**FIGURE 1:**
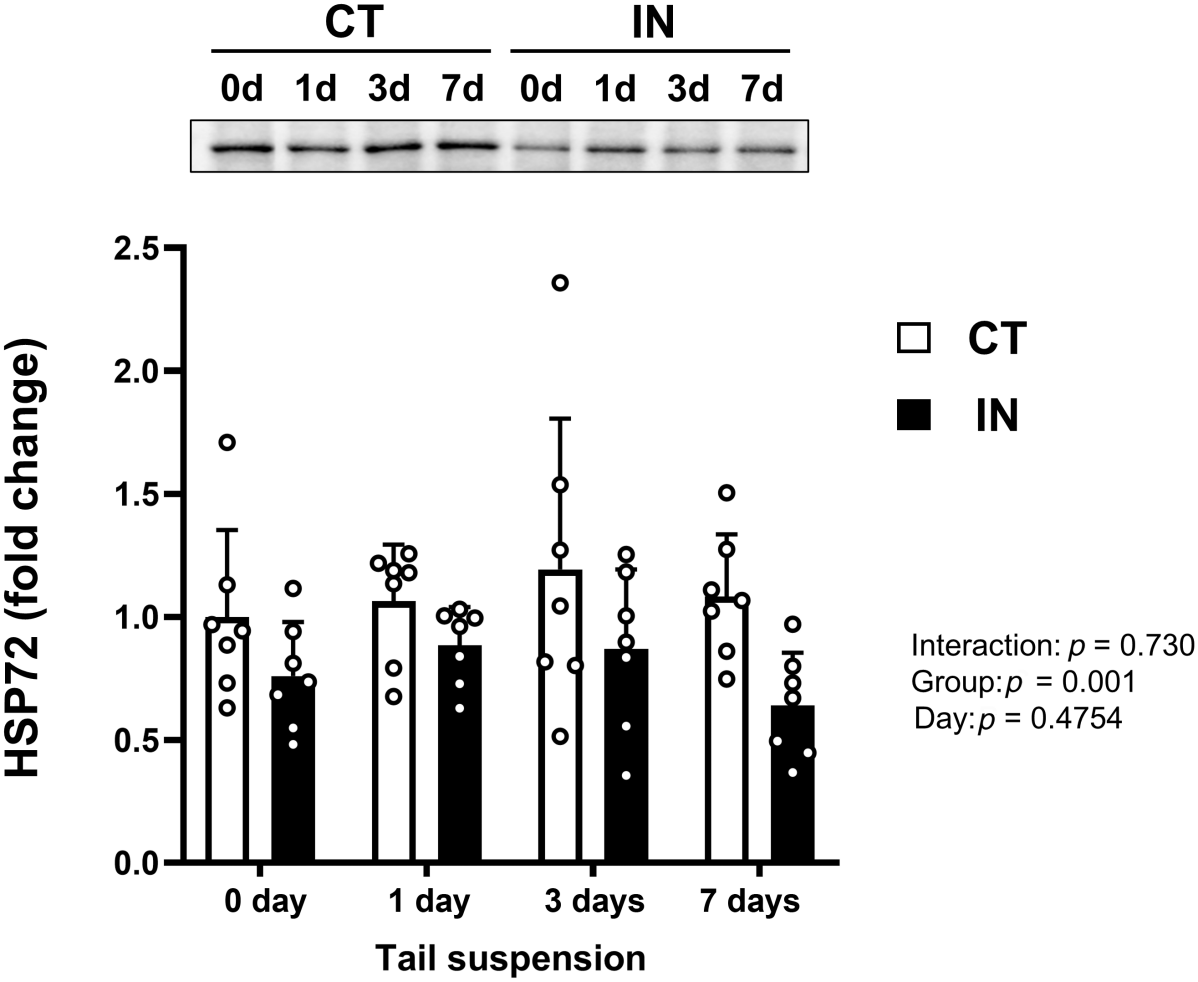
HSP72 protein expression levels after 7 days (7d) of tail suspension. Samples were collected before (0 day, 0d; immediately after 8 weeks of intervention), and at 1 day (1d), 3 days (3d), and 7 days (7d) after tail suspension (*n* = 6–7 per group). Values are means ± SD. The results of the two‐way analysis of variance are shown. The expression level of each protein was normalized to the total protein loading. CT, control group; IN, inactivity group.

To investigate whether long‐term physical inactivity during adolescence decreases cytoprotective and anti‐apoptotic protein expressions, we initially evaluated the hepatic HSP72 expression levels (Figure 1). The IN group had significantly lower HSP72 levels compared to the CT group over 7 days of tail suspension (*p* = 0.001), whereas tail suspension itself did not have a significant impact on HSP72 levels (*p* = 0.475).

### 3.3 Fragmented nucleosomes

**FIGURE 2:**
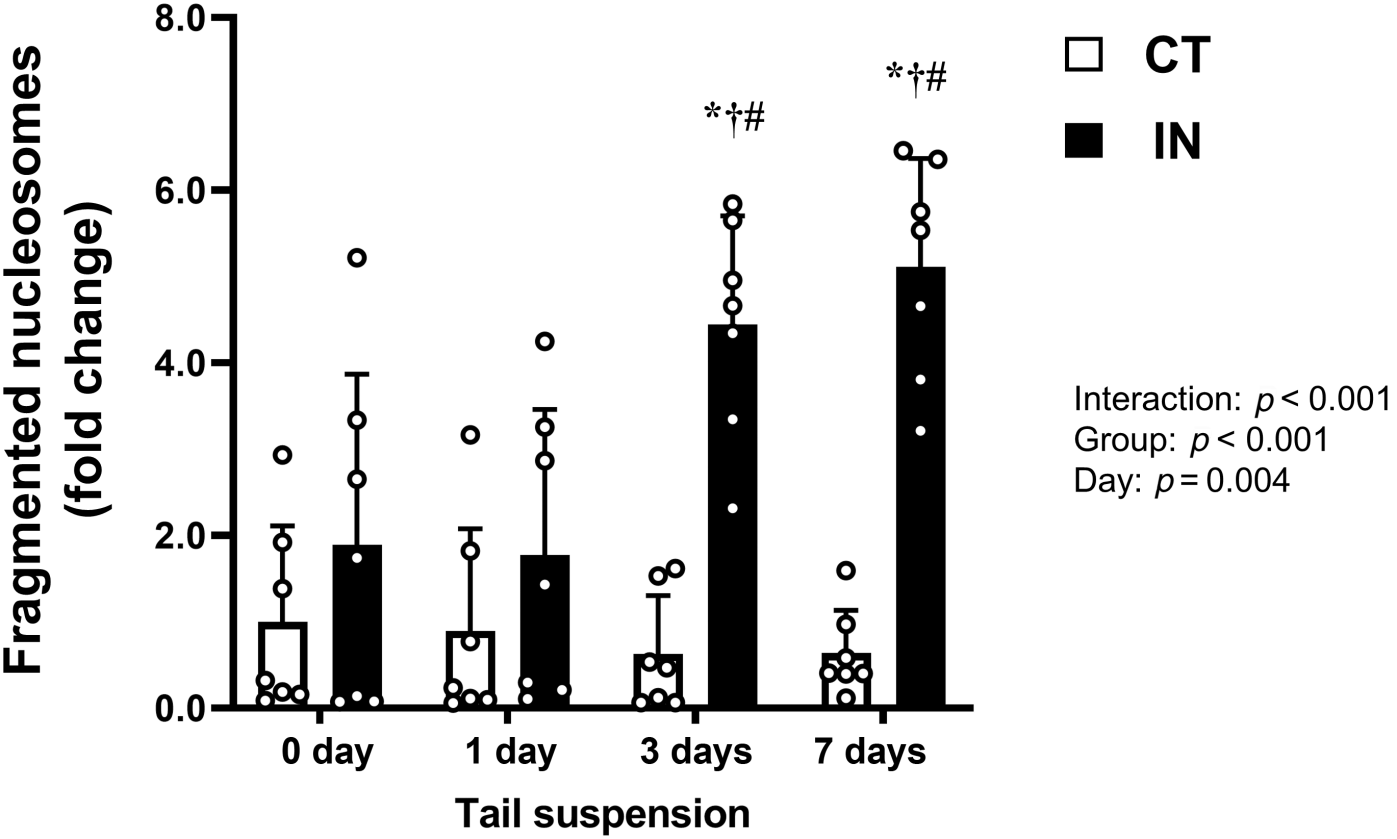
Fragmented nucleosomes after 7 days (7d) of tail suspension. Values are means ± SD. The results of the two‐way analysis of variance are displayed. *p < 0.0001 versus CT at same day (3 days [3d] and 7 days [7d]), ^†^ *p* < 0.05 versus IN at 0d, ^#^ *p* < 0.01 versus IN at 1 day (1d). CT, control group; IN, inactivity group.

Since the physical inactivity intervention decreased hepatic HSP72 expression and its lower levels lasted throughout the 7 days of tail suspension, we next assessed cytoplasmic fragmented nucleosomes as an apoptotic index in the liver (Figure 2). A significant interaction between the group and day was observed (*p* < 0.001). Although the level of fragmented nucleosomes in the CT group did not change during tail suspension, the levels in the IN group gradually and drastically increased. At 3 and 7 days after tail suspension, the fragmented nucleosomes in the IN group were significantly greater than those in the CT group (*p* < 0.001).

### 3.4 Cleaved caspase‐3 and ‐7 expression levels

**FIGURE 3:**
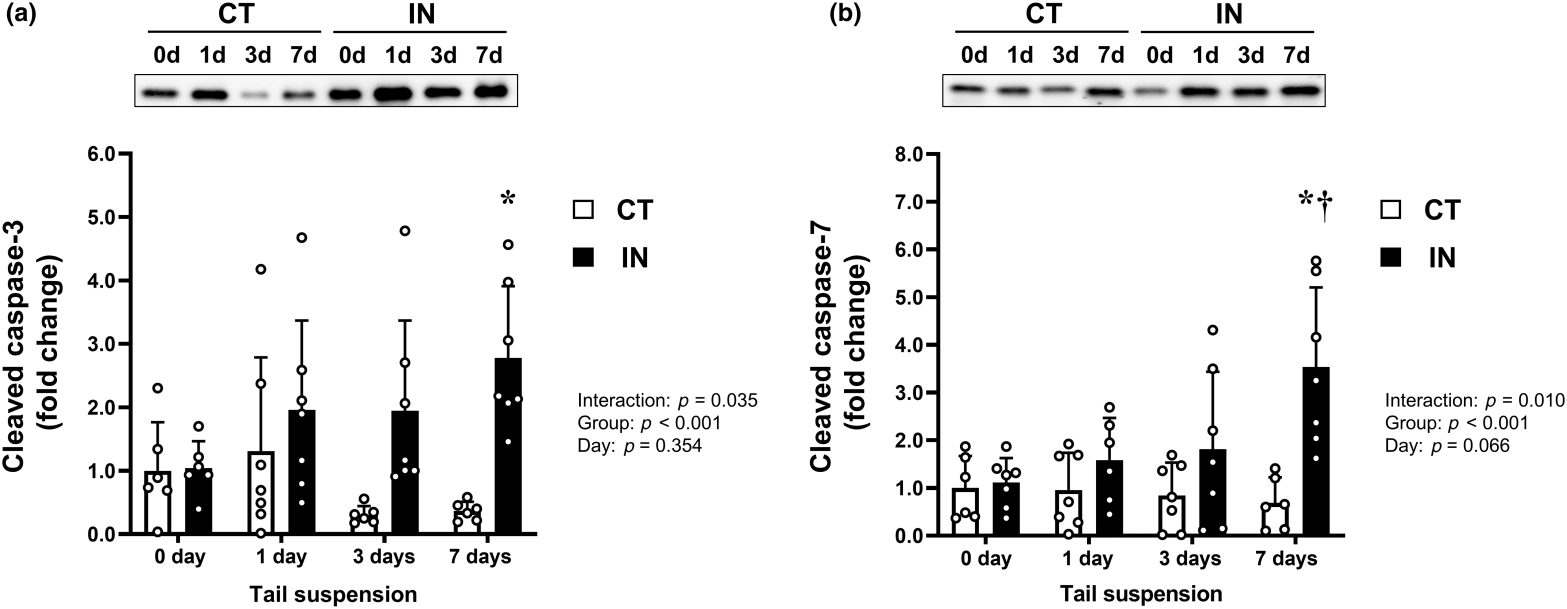
Cleaved caspase‐3 (a) and ‐7 (b) protein expression levels after 7 days (7d) of tail suspension. Values are means ± SD. The results of two‐way analysis of variance are displayed. The expression level of each protein was normalized to the total protein loaded. **p < 0.001 versus CT at 7 days (7d), ^†^ *p* < 0.05 vs. IN at 0 day (0d). CT, control group; IN, inactivity group.

To address the underlying mechanisms of the upregulation of hepatic apoptosis by physical inactivity and tail suspension, we determined the protein expression levels of cleaved caspase‐3 and ‐7 during tail suspension (Figure 3). For both cleaved caspase protein expressions, significant interactions between group and day were observed (*p* = 0.035 for cleaved caspase‐3, *p* = 0.010 for cleaved caspase‐7). Levels of both cleaved caspase proteins at 7 days after tail suspension were significantly higher in the IN group than in the CT group (both *p* < 0.01).

### 3.5 TNF‐α expression levels

**FIGURE 4:**
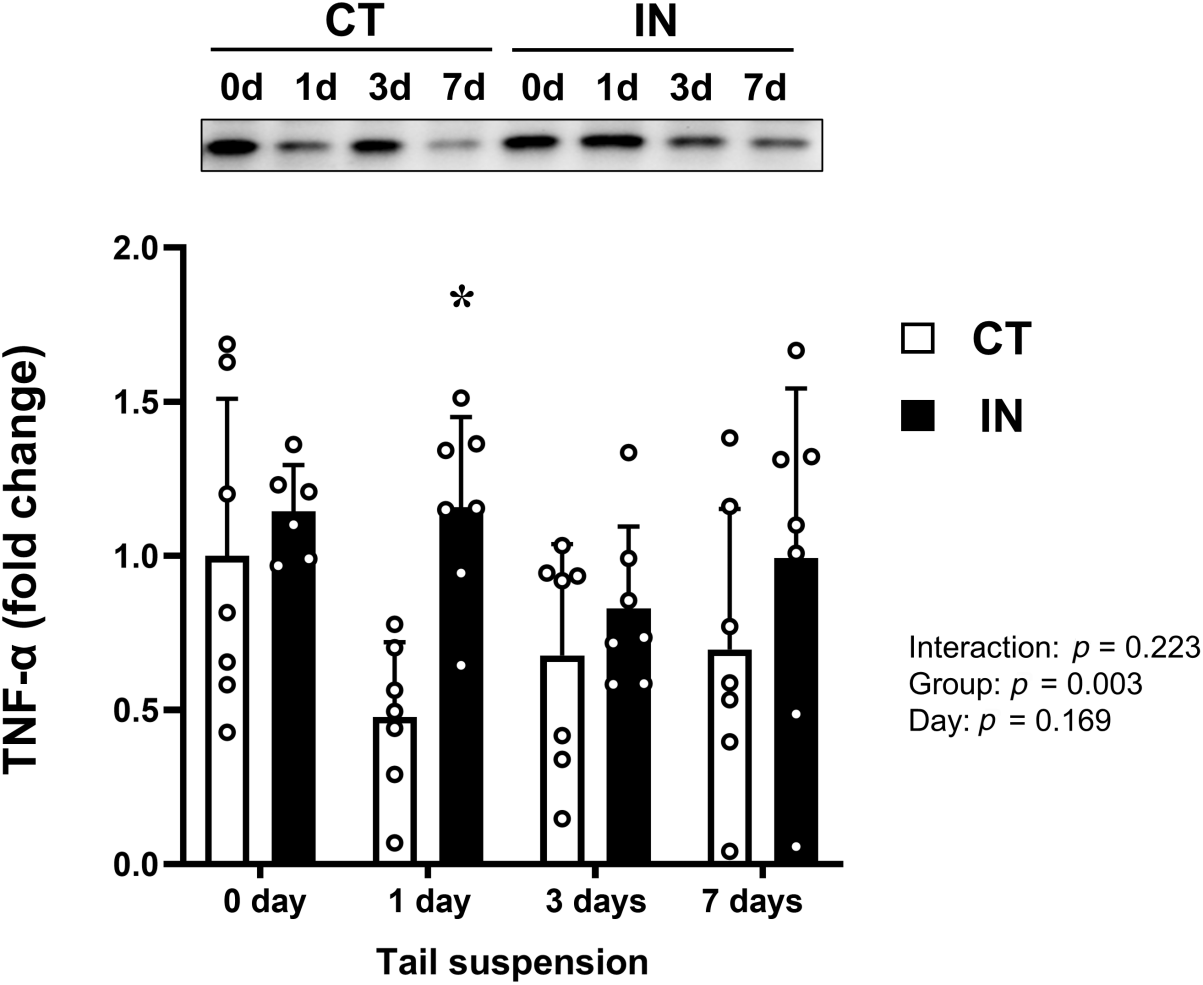
TNF‐α protein expression levels after 7 days (7d) of tail suspension. Values are means ± SD. The results of the two‐way analysis of variance are displayed. The expression level of each protein was normalized to the total protein loaded. *p < 0.05 versus CT at 1 day (1d). CT, control group; IN, inactivity group.

To assess the index of promoting hepatic inflammation and cell death, we quantified the protein levels of TNF‐α in the liver (Figure 4). A significant main effect of the group was detected in TNF‐α levels (*p* = 0.003), which were significantly higher in the IN group than in the CT group 1 day after tail suspension (*p* < 0.05).

### 3.6 HDACs 4 and 5 expression levels

**FIGURE 5:**
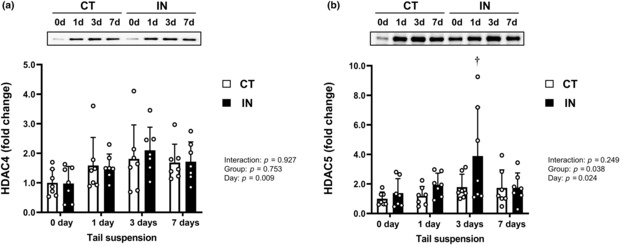
Protein expression levels of HDAC4 (a) and HDAC5 (b) after 7 days (7d) of tail suspension. Values are means ± SD. The results of two‐way analysis of variance are displayed. The expression level of each protein was normalized to the total protein loaded. ^†^ *p* < 0.05 versus IN at 0 day (0d). CT, control group; IN, inactivity group.

HDACs 4 and 5 are associated with liver injury and the development of hepatocellular carcinoma (Freese et al., 2019), and we previously found greater HDAC4 expression in rat soleus muscle during tail suspension after 8‐week physical inactivity intervention (Yoshihara et al., 2021). Therefore, we investigated the expression levels of HDACs 4 and 5 in the liver during tail suspension (Figure 5). While physical inactivity during adolescence did not affect HDAC4 expression, tail suspension increased it (*p* = 0.009; Figure 5a). We found significant main effects of group and day (group: *p* = 0.038; day: *p* = 0.024) on HDAC5 levels, and these levels in the IN group were significantly elevated 3 days after tail suspension compared with levels at 0 day (*p* < 0.05; Figure 5b).

### 3.7 LC3 and p62 expression levels

**FIGURE 6:**
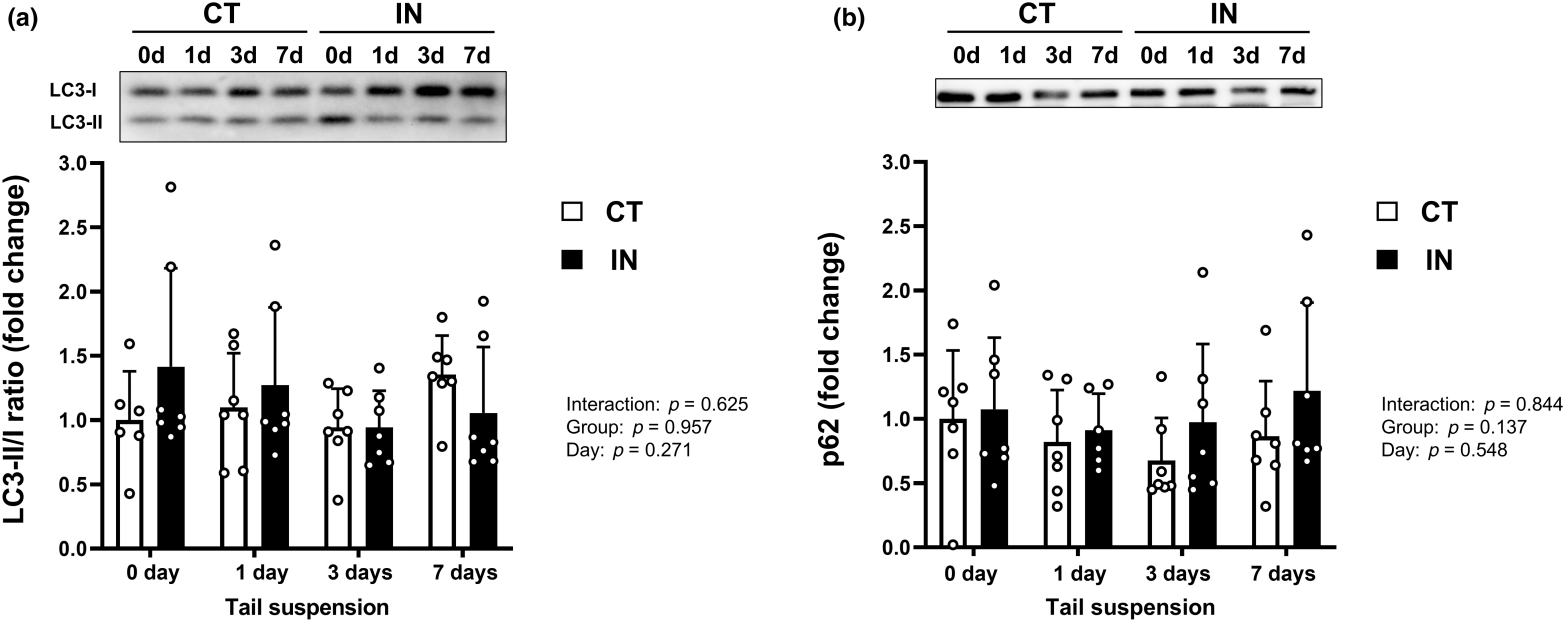
LC3‐II/I ratio (a) and p62 protein expression levels (b) after 7 days (7d) of tail suspension. Values are means ± SD. The results of two‐way analysis of variance are displayed. The expression level of p62 protein was normalized to the total protein loaded. CT, control group; IN, inactivity group.

We also measured hepatic autophagy‐related protein expression levels (LC3‐II/I ratio and p62; Figure 6). These autophagic markers did not change because of physical inactivity or tail suspension.

### 3.8 Correlation between hepatic HSP72 and apoptosis indicators

As we hypothesized that the reduction in HSP72 levels by physical inactivity is related to accelerated hepatic apoptosis during tail suspension via increasing pro‐apoptotic protein expression, we conducted a correlation analysis between hepatic HSP72 levels and indicators of apoptosis (i.e., cleaved caspase‐3 and ‐7, TNF‐α, HDACs 4 and 5, and fragmented nucleosome) (Table 1). Hepatic HSP72 expression was significantly correlated with cleaved caspase‐3 (r_s_ = −0.296, *p* = 0.033) and ‐7 (r_s_ = −0.289, *p* = 0.036), and TNF‐α (r_s_ = −0.326, *p* = 0.015), and marginally correlated with the levels of fragmented nucleosomes (r_s_ = −0.247, *p* = 0.067). However, no correlation was observed between HSP72 and the expression of HDACs 4 and 5 (both *p* > 0.05).

**TABLE 1:** Spearman's correlation coefficient between hepatic HSP72 levels and pro‐apoptotic proteins and fragmented nucleosomes.

	r_s_‐value	*p*‐value
Cleaved caspase‐3	−0.296	0.033
Cleaved caspase‐7	−0.289	0.036
TNF‐α	−0.326	0.015
HDAC4	0.041	n.s.
HDAC5	0.068	n.s.
Fragmented nucleosome	−0.247	0.067

*Note*: n.s., not significant.

## 4 DISCUSSION

This study investigated the impact of long‐term physical inactivity on hepatic cytoprotective‐ and inflammatory‐related protein expressions and the subsequent apoptotic responses during tail suspension in young rats. We demonstrated that tail suspension following 8 weeks of physical inactivity drastically increased the levels of fragmented nucleosomes in the cytoplasmic fraction of the liver. Although the precise mechanism underlying these phenomena is unknown, our findings suggest that decreased HSP72 levels by physical inactivity might promote subsequent hepatic apoptosis during tail suspension by increasing pro‐apoptotic proteins (cleaved caspase‐3 and ‐7, and TNF‐α).

Regular physical activity is known to contribute to maintaining hepatic health by attenuating inflammation and apoptosis, and improving metabolism (Zelber‐Sagi et al., 2021); the underlying mechanisms might involve the upregulation of hepatic HSP72 expression induced by exercise training (Henstridge et al., 2016). A previous systematic review of epidemiological studies demonstrated that physical inactivity adversely affects hepatic health (Zelber‐Sagi et al., 2021). However, it is unclear whether a chronic sedentary lifestyle alters HSP72 levels in the liver. Thus, we initially assessed hepatic HSP72 levels after 8 weeks of physical inactivity and found that long‐term physical inactivity reduced the hepatic HSP72 expression levels. In contrast, HSP72 levels in the liver did not change during the 7‐day tail suspension. Notably, a previous study demonstrated that hepatic HSP72 levels were increased at 6 hours of tail suspension, but transiently decreased to baseline levels within 12 hours (Cui et al., 2010). This evidence can support our results. Thus, a decrease in HSP72 levels may be only observed because of physical inactivity. As HSP72 has various cytoprotective properties in the liver (Balchin et al., 2016), our data suggest that physical inactivity might reduce hepatic homeostatic systems.

A recent narrative review of previous experimental studies suggests that tail suspension induces inflammation and apoptotic responses in the liver (Prasad et al., 2020). In this study, we demonstrated that physical inactivity at a young age exacerbated the subsequent hepatic fragmented nucleosomes during tail suspension. While previous studies have demonstrated that a relatively long‐term (>14 days) tail suspension causes hepatic apoptosis and inflammation (Chen et al., 2017; Du et al., 2015; Rivera et al., 2003), the relatively short‐term tail suspension in this study (7 days) might not cause hepatic apoptosis in the CT group. Moreover, as a previous review demonstrated that apoptosis itself does not lead to increased inflammation (Prasad et al., 2020), TNF‐α levels might not be changed by tail suspension. However, our findings suggest that 8 weeks of physical inactivity accelerated hepatic apoptosis during tail suspension. Regarding the mechanism underlying the acceleration of hepatic apoptosis by physical inactivity, we found that the major pro‐apoptotic proteins (i.e., cleaved caspase‐3 and ‐7) were synergistically increased in the inactive rats. Moreover, TNF‐α levels were significantly higher in the IN group than in the CT group over 7 days of tail suspension. TNF‐α and tail suspension stimulate the activation of cleaved caspase‐3 and ensuing apoptosis in the liver (Du et al., 2015; Leveille et al., 2020). These findings suggest that an increase in inflammation (TNF‐α) caused by physical inactivity may be linked to the promotion of hepatic apoptosis via increasing the expression levels of cleaved caspases during tail suspension. Further, both cleaved caspases and TNF‐α are attenuated by the upregulation of HSP72 expression (Tang et al., 2007; Tsai et al., 2015); indeed, we confirmed significant negative relationships between hepatic HSP72 and these pro‐apoptotic proteins in this study. Therefore, the reduction in hepatic HSP72 levels by long‐term physical inactivity might enhance hepatic apoptosis during tail suspension by increasing the levels of these pro‐apoptotic proteins, although cause‐and‐effect relationships are still unknown.

Recently, it has been suggested that HDACs 4 and 5 expression levels are associated with liver injury and the development of hepatocellular carcinoma (Freese et al., 2019) and hepatic fibrosis (Van Beneden et al., 2013). Accordingly, we assessed the hepatic HDACs 4 and 5 levels during tail suspension after long‐term physical inactivity. Tail suspension significantly increased hepatic HDACs 4 and 5 expressions, and physical inactivity had a significant impact on the response of hepatic HDAC5 expression to unloading. We also evaluated hepatic α‐smooth muscle actin level, an HDACs downstream marker involved in inducing hepatic inflammation and fibrosis, and the change in the protein expression was not associated with the change in the HDACs expression (data not shown). Since previous studies suggest that the upregulation of hepatic HDAC levels by physical inactivity and tail suspension might be an initial critical step in the deleterious impact on hepatic homeostasis (Van Beneden et al., 2013), the physiological significance of the increase in HDAC levels currently remains unknown. Moreover, as HDAC inhibitor‐induced hepatoprotective effects against ischemia–reperfusion injury are associated with increased HSP72 expression (Sun et al., 2014), we assumed that the upregulation of HDAC5 levels might be partly related to the physical inactivity‐induced reduction of HSP72 levels. However, no significant correlation between hepatic HSP72 and HDAC5 levels was found in this study. Thus, the mechanisms underlying the increased level of hepatic HDAC5 by physical inactivity should be investigated in more detail.

A previous study suggested that exercise training attenuates the hepatic apoptotic response by increasing autophagic capacity (Kwon et al., 2020); therefore, we also evaluated the LC3‐II/I ratio and p62 protein levels in the liver. However, both the LC3‐II/I ratio and p62 protein levels were not altered by physical inactivity or tail suspension. This result indicates that the impact of autophagic responses to physical inactivity and tail suspension on hepatic apoptosis might be small.

Our findings demonstrated that long‐term physical inactivity during adolescence had a negative impact on hepatic health. We previously observed that physical inactivity at a young age exacerbated subsequent hindlimb unloading‐induced atrophy in the rat soleus muscle (Yoshihara et al., 2021). Therefore, we provide insights that regular physical activity in childhood is important for life‐long health. However, we did not perform a histological analysis of hepatic phenotype owing to the methodological limitation of sample preparation and thus, could not demonstrate the detailed mechanisms underlying how long‐term physical inactivity downregulates hepatic HSP72 expression. Physical activity can alter epigenetic regulation, such as histone modification, DNA methylation, and microRNA expression, in various organs, including the liver (Stevanovic et al., 2020), which may be related to the reduction in HSP72 levels by physical inactivity. Previous studies have indicated that apoptosis may be involved in several age‐related liver disorders including nonalcoholic fatty liver disease, liver fibrosis, cirrhosis, and liver cancer (Hu et al., 2019; Shojaie et al., 2020); however, we did not evaluate hepatic function in this study. Therefore, future studies with expanded measurements to clarify the detailed mechanisms of physical inactivity‐induced hepatic dysfunction are warranted.

In conclusion, our data suggest that 8 weeks of physical inactivity accelerate subsequent hepatic apoptosis during tail suspension, possibly via decreased hepatic HSP72 levels and increased pro‐apoptotic proteins in young male rats.

## AUTHOR CONTRIBUTIONS

Conception and design of the experiments: Shohei Dobashi, Hisashi Naito, and Toshinori Yoshihara. Collection, analysis, and interpretation of data: Shohei Dobashi and Toshinori Yoshihara. All authors drafted the article or revised it critically for important intellectual content. All authors have read and approved the final version of this manuscript and agree to be accountable for all aspects of the work in ensuring that questions related to the accuracy or integrity of any part of the work are appropriately investigated and resolved. All persons designated as authors qualify for authorship, and all those who qualify for authorship are listed.

## FUNDING INFORMATION

This work was supported by the Japan Society for the Promotion of Science KAKENHI (Grant no. 21K19735 and 17K01765 to T. Yoshihara). This work was also supported by the Institute of Health and Sports Science & Medicine, Juntendo University.

## CONFLICT OF INTEREST STATEMENT

All authors declare no conflicts of interest.
